# Supplementary material for: Symmetry broken and rebroken during the ATP hydrolysis cycle of the mitochondrial Hsp90 TRAP1
Source: eLife. 2017 Jul 25;6:e25235. doi: 10.7554/eLife.25235 (PMC5550277; doi:10.7554/eLife.25235)
Supplement: Supplementary file 1 — Each dataset was collected from a single crystal. Values in parentheses are for highest-resolution shell. DOI: http://dx.doi.org/10.7554/eLife.25235.014 [file elife-25235-supp1.docx]

**Supplementary file 1A.** **Data collection and refinement statistics (molecular replacement)**

|  | **zTRAP1 +/R417A** | **WT zTRAP1**  **Day 1** | **WT zTRAP1**  **Day 3** | **WT zTRAP1**  **Day 53** |
| --- | --- | --- | --- | --- |
| **Data collection** |  |  |  |  |
| Space group | C 1 2 1 | C 1 2 1 | C 1 2 1 | C 1 2 1 |
| Cell dimensions |  |  |  |  |
| *a*, *b*, *c* (Å) | 180.086, 95.711, 126.558 | 175.881, 96.9902, 124.961, | 178.49, 97.181, 125.904 | 178.491, 96.82, 125.78 |
| α, β, γ (°) | 90.0, 134.592, 90.0 | 90, 134.568, 90 | 90, 134.641, 90 | 90, 134.18, 90 |
| Resolution (Å) | 49.0 - 3.20 | 44.52 - 2.3 | 42.67 - 2.14 | 40.54 - 1.99 |
| *R*_sym_ or *R*_merge_ | 0.146 | 0.516 | 0.043 | 0.055 |
| *I* / σ*I* | 9.1 (1.0) | 2.2 (0.29) | 11 (0.76) | 7.8 (0.29) |
| Completeness (%) | 83.03 | 93.4 | 99.5 | 99.6 |
| Redundancy | 2.5 | 1.8 | 1.9 | 1.9 |
|  |  |  |  |  |
| **Refinement** |  |  |  |  |
| Resolution (Å) | 49.0 -3.20 | 39.67 - 3.5 | 39.89 - 2.5 | 39.97 - 2.2 |
| No. reflections | 21134 (2095) | 18821 (1856) | 98531 (9720) | 77629 (7714) |
| *R*_work_ / *R*_free_ | 0.213 / 0.257 | 0.1977 / 0.2558 | 0.1804 / 0.2377 | 0.1885 / 0.2365 |
| No. atoms |  |  |  |  |
| Protein | 10383 | 9592 | 9597 | 9570 |
| Ligand/ion | 66 | 62 | 66 | 62 |
| Water | - |  |  |  |
| *B*-factors |  |  |  |  |
| Protein | 87.70 | 72.29 | 68.56 | 66.32 |
| Ligand/ion | 59.90 | 72.36 | 45.99 | 69.99 |
| Water | - | 62.38 | 52.91 | 55.34 |
| R.m.s. deviations |  |  |  |  |
| Bond lengths (Å) | 0.0122 | 0.003 | 0.008 | 0.007 |
| Bond angles (°) | 1.4721 | 0.604 | 1.01 | 0.96 |

*Each dataset was collected from a single crystal.

*Values in parentheses are for highest-resolution shell.
